# Genetic Ablation of the Mitochondrial Calcium Uniporter (MCU) Does not Impair T Cell-Mediated Immunity *In Vivo*

**DOI:** 10.3389/fphar.2021.734078

**Published:** 2021-12-20

**Authors:** Hao Wu, Benjamin Brand, Miriam Eckstein, Sophia M. Hochrein, Magdalena Shumanska, Jan Dudek, Alexander Nickel, Christoph Maack, Ivan Bogeski, Martin Vaeth

**Affiliations:** ^1^ Würzburg Institute of Systems Immunology, Max Planck Research Group at the Julius-Maximilians-Universität Würzburg, Würzburg, Germany; ^2^ Molecular Physiology, Institute of Cardiovascular Physiology, University Medical Center, Georg-August-University, Göttingen, Germany; ^3^ Comprehensive Heart Failure Center (CHFC), University Hospital, Julius-Maximilians University of Würzburg, Würzburg, Germany

**Keywords:** mitochondrial calcium uniporter (MCU), store-operated Ca^2+^ entry, mitochondria, oxidative phosphorylation, calcium (Ca^2+^), immunometabolism, mitochondrial Ca^2+^ handling

## Abstract

T cell activation and differentiation is associated with metabolic reprogramming to cope with the increased bioenergetic demand and to provide metabolic intermediates for the biosynthesis of building blocks. Antigen receptor stimulation not only promotes the metabolic switch of lymphocytes but also triggers the uptake of calcium (Ca^2+^) from the cytosol into the mitochondrial matrix. Whether mitochondrial Ca^2+^ influx through the mitochondrial Ca^2+^ uniporter (MCU) controls T cell metabolism and effector function remained, however, enigmatic. Using mice with T cell-specific deletion of MCU, we here show that genetic inactivation of mitochondrial Ca^2+^ uptake increased cytosolic Ca^2+^ levels following antigen receptor stimulation and store-operated Ca^2+^ entry (SOCE). However, ablation of MCU and the elevation of cytosolic Ca^2+^ did not affect mitochondrial respiration, differentiation and effector function of inflammatory and regulatory T cell subsets *in vitro* and in animal models of T cell-mediated autoimmunity and viral infection. These data suggest that MCU-mediated mitochondrial Ca^2+^ uptake is largely dispensable for murine T cell function. Our study has also important technical implications. Previous studies relied mostly on pharmacological inhibition or transient knockdown of mitochondrial Ca^2+^ uptake, but our results using mice with genetic deletion of MCU did not recapitulate these findings. The discrepancy of our study to previous reports hint at compensatory mechanisms in MCU-deficient mice and/or off-target effects of current MCU inhibitors.

## Introduction

Mitochondria play a pivotal role in cellular metabolism by producing large amounts of ATP through oxidative phosphorylation (OXPHOS) and fatty acid oxidation (FAO). In addition, intermediates of mitochondrial metabolism, such as tricarboxylic acid (TCA) cycle, glutaminolysis and FAO, control the fate and function of immune cells by regulating signaling pathways, the cellular redox balance, apoptosis and epigenetic rewiring through DNA and histone modifications. Not surprisingly, mitochondrial metabolism has been (re-)discovered as an important regulator of lymphocyte function and “immunometabolism” has become a major research focus.

The observation that mitochondria can rapidly take up calcium (Ca^2+^) from the cytosol was made over half a century ago (Finkel et al., 2015). Studies in myocytes and other non-immune cells demonstrated that mitochondrial Ca^2+^ handling can regulate mitochondrial metabolism and function, including the activity of the TCA cycle and the electron transport chain (ETC), the production of reactive oxygen species (ROS) and apoptosis through opening of the mitochondrial permeability transition pore (mPTP) (Finkel et al., 2015; Wang P. et al., 2020). Although the outer mitochondrial membrane is permeable to most inorganic ions, transport of Ca^2+^ at the inner mitochondrial membrane (IMM) is tightly regulated and requires specific transporters. Following an intensive search over decades, two groups identified the predicted *mitochondrial Ca* ^*2+*^ *uniporter* (MCU) using an *in silico* approach and demonstrated that the MCU protein forms a highly selective, inwardly rectifying Ca^2+^ channel. After the molecular identification of MCU, it became clear that mitochondrial Ca^2+^ uptake is mediated by a larger protein complex that not only contains MCU as the pore-forming subunit but is composed of additional regulatory and structural elements. *Essential MCU regulator* (EMRE) and its homologue MCUb were shown to be part of the channel pore, whereas the two EF hand domain-containing proteins *mitochondrial Ca* ^*2+*^ *uptake 1* (MICU1), 2 and 3 function as “gatekeepers” of the MCU complex (Wang P. et al., 2020).

Ca^2+^ influx into the mitochondrial matrix is driven by a negatively charged membrane potential at the IMM that is established by the ETC. The opening of the MCU channel is initiated by conformational changes of MICU1/2 after binding of free cytosolic Ca^2+^ to their EF hand domains. Given the relatively low affinity of MICU1/2 for Ca^2+^ it was originally believed that the extramitochondrial Ca^2+^ levels required for MCU activation could not be achieved under physiological conditions. However, most Ca^2+^ signals are not uniformly distributed throughout the cytosol but are locally restricted to the site of Ca^2+^ influx. These Ca^2+^ “microdomains” can be generated by Ca^2+^ release from ER stores and/or extracellular calcium influx pathways, such as *store-operated Ca* ^*2+*^ *entry* (SOCE) through *Ca* ^*2+*^ *release-activated Ca* ^*2+*^ (CRAC) channels, and reach the required local Ca^2+^ concentration to induce MCU channel opening. In activated T cells, mitochondria are in proximity to the ER and move actively towards the immunological synapse at which Ca^2+^ influx *via* SOCE is elicited (Quintana et al., 2007; Lioudyno et al., 2008; Quintana et al., 2011). We showed recently that CRAC channels and SOCE not only control mitochondrial size and function but also mitochondrial metabolism, including TCA cycle and OXPHOS (Vaeth et al., 2017a; Kahlfuss et al., 2020). These effects could be explained by a direct, MCU-mediated uptake of cytosolic Ca^2+^ into the mitochondrial matrix and the positive regulation of Ca^2+^-sensitive TCA cycle enzymes. On the other hand, SOCE may also mediate these effects indirectly by regulating gene expression of nuclear-encoded mitochondrial proteins. To clarify how MCU-dependent mitochondrial Ca^2+^ uptake contributes to T cell metabolism and function *in vivo*, we investigated mice with genetic deletion of MCU in T cells using models of autoimmunity and viral infection.

## Materials and Methods

### Mice

*Mcu* ^fl/fl^ (Kwong et al., 2015) (strain 029817), *Foxp3* ^Cre^ (strain 016959) and *Cd4* ^Cre^ mice (strain 022071) were purchased from the Jackson laboratories and housed at the University of Würzburg. All animals were on a pure C57BL/6J genetic background and maintained under SPF conditions.

### T cell cultures and treatments

T cells were isolated using MojoSort Mouse T cell isolation kits (BioLegend) and cultured in complete RPMI 1640 medium (Gibco) containing 1 g/L glucose. 24-well plates were pre-coated with 12 μg/ml polyclonal anti-hamster IgG, washed with PBS and activated with either 0.25 μg/ml (for Th17) or 0.5 μg/ml (for Th1 and iTreg subsets) of anti-CD3 (clone 145-2C1) together with 1 μg/ml anti-CD28 (37.51). The following culture conditions were used: Th1 cells: 2.5 μg/ml anti-IL-4 (clone 11B11), 10 ng/ml rhIL-2 and 10 ng/ml rmIL-12. iTreg cells: 2.5 μg/ml anti-IL-4, 2.5 μg/ml anti-IFNγ, 10 ng/ml rhIL-2 and 5 ng/ml rhTGF-beta. Th17 cells: 2.5 μg/ml anti-IL-4, 2.5 μg/ml anti-IFNγ, 20 ng/ml rmIL-6 and 0.5 ng/ml rhTGFβ. Tc1 cells: 2.5 μg/ml anti-IL-4, 10 ng/ml rhIL-2 and 10 ng/ml rmIL-12. Tc17 cells: 2.5 μg/ml anti-IL-4, 2.5 μg/ml anti-IFNγ, 20 ng/ml rmIL-6 and 0.5 ng/ml rhTGFβ. All antibodies and cytokines were from Bio X Cell and Peprotech, respectively. To detect cytokine expression, cells were stimulated with 1 μM ionomycin plus 30 nM phorbol myristate acetate (Calbiochem) for 5 h in the presence of brefeldin A (BioLegend). In some experiments, T cell were treated for 72 h with 2–10 µM Ruthenium red (Sigma).

### Experimental Autoimmune Encephalomyelitis

MOG_35–55_ peptide (Synpeptide) was emulsified in CFA with 5 mg/ml *M tuberculosis* H37Ra (BD Difco). 200 mg MOG_35–55_ was injected subcutaneously at the flanks of the mice, followed by intraperitoneal injection of 250 ng pertussis toxin (Enzo) on day 0 and 2. The EAE score was assigned as described before (Stromnes and Goverman, 2006). Mice were sacrificed and spinal cords were isolated, minced into small pieces and digested with 1 mg/ml collagenase D (Roche) and 20 μg/ml DNase I (Sigma) for 40 min at 37°C. CNS-infiltrating lymphocytes were enriched by percoll gradient centrifugation and analyzed by flow cytometry.

### LCMV Infections

The LCMV clone 13 strain was kindly provided by R. Ahmed (Emory University). LCMV was grown in BHK-21 cells and viral titers (PFU) in the supernatant were determined as described (Vaeth et al., 2016; Ataide et al., 2020). For chronic viral infections, mice were injected intravenously with 4 × 10^6^ PFU of LCMV and analyzed 23 days post infection.

### Flow Cytometry

Cells were blocked with anti-FcγRII/FcγRIII (Bio X Cell; clone 2.4G2) and debris was excluded using the viability dye eFluor780 (eBioscience). Staining of surface molecules with fluorochrome-conjugated antibodies was performed in PBS containing 0.1% BSA. Intracellular (IC) and transcription factor (TF) staining was carried out with the IC Staining and TF Staining Buffer Kit, respectively (eBioscience). Samples were acquired on a BD FACSCelesta flow cytometer and analyzed with FlowJo Software (Tree Star). The following antibodies were used: anti-mouse CD4 (clones 53–6.7 and GK1.5), anti-mouse IL-17A (C11-18H10.1), anti-mouse IFNγ (XMG1.2), anti-mouse GM-CSF (MP1-22E9), anti-mouse TNFα (MP6-XT22), anti-mouse IL-2 (JES6-5H4), anti-mouse Foxp3 (FJK-16s), anti-mouse T-bet (4B10), anti-mouse RORγt (Q31-378), anti-mouse CD25 (PC61), anti-mouse Ki-67 (B56), anti-CD44 (IM7), anti-CXCR5 (SPRCL5), anti-PD-1 (RMP1-30), anti-CD38 (90), anti-GL.7 (GL7), anti-CD8α (53–6.7), anti-Tim3 (RMT3-23). All antibodies were from BioLegend or eBiosciences. PE-labelled tetramers loaded with the LCMV peptides GP_33-41_ and GP_66-77_ were provided by the NIH Tetramer Core Facility.

### T Cell Proliferation Analysis

T cells were labelled with CellTrace Violet (Thermo Scientific) according to the manufacturer’s instructions. Cells were blocked with 50% FBS, washed twice with RPMI and stimulated with 1 μg/ml plate-bound anti-CD3 (clone 2C11) and anti-CD28 antibodies (37.51; both Bio X Cell) with 50 U/ml IL-2 (Peprotech). CTV dilution was monitored daily by flow cytometry.

### Ca^2+^ Influx Measurements

T cells were labelled with 2 μM Fura-2-AM (Thermo Scientific) as described earlier (Vaeth et al., 2017b). Cells were attached to 96-well imaging plates coated with 0.01% poly-L-lysine (Sigma) and washed with Ca^2+^-free Ringer solution. Changes in intracellular Ca^2+^ concentration were analyzed using a FlexStation3 plate reader (Molecular Devices) at 340 and 380 nm. Cells were stimulated with 1 μM thapsigargin (TG) (Sigma) in Ca^2+^-free Ringer solution and SOCE was analyzed after re-addition of 2 mM Ca^2+^. Alternatively, whole splenocytes were loaded with 2 μM Fluo4-AM (Thermo Scientific) and incubated with anti-CD4-APC and anti-CD8a-BV421 antibodies (BioLegend). Measurements of intracellular Ca^2+^ changes were recorded by flow cytometry. Baseline cytosolic Ca^2+^ levels acquired in 0 mM Ca^2+^ Ringer solution, before cells were stimulated with 1 μM thapsigargin and re-addition of 2 mM Ca^2+^. Mitochondrial uptake of free Ca^2+^ was monitored in the presence of digitonin-permeabilized T cells (0.04%; preincubation time 10 min). 1 × 10^6^ cells per well were re-suspended in PTP buffer (125 mM KCl, 2 mM K_2_HPO_4_, 1 mM MgCl_2_, 10 mM HEPES, 20 µM EGTA, pH 7.4) containing 2 mM succinate, 2 µM thapsigargin and 1 μM CalciumGreen-5N (Thermo Scientific). Fluorescence (503/535 nm) was monitored using a Tecan M200 Pro reader in a 96-well plate with injection of 25 µM Ca^2+^ pulses.

### Histochemistry

CNS specimens were fixed in 4% PFA, embedded in paraffin and stained with Luxol fast blue and Cresyl violet (both Carl Roth) to detect myelin and nuclei, respectively.

### Quantitative RT-PCR

RNA was extracted using the RotiPrep RNA mini kit (Carl Roth), followed by cDNA synthesis with the iScript cDNA synthesis Kit (BioRad). qRT-PCR was performed using iTaq Universal SYBR Green SuperMix (Bio-Rad) and the CFX RT-PCR thermocycler (BioRad). For quantitation, *C*_T_ values were normalized to *18S* gene expression and analyzed using the 2^−ΔCT^ method. *Mcu-*for: TTA​GCA​GAA​AAG​CAG​AGA​AGA​G, *Mcu*-rev: TGA​TGA​AGT​AGG​TGA​CGG​G, *Letm1*-for: CGG​GGT​AGT​CTG​AGG​GAT​CG, *Letm1*-rev: TGG​AGT​ACA​GCA​ACG​AGA​CAG, *Slc8b1*-for: CTG​GAA​GTG​TCA​ACC​AGA​CTG, *Slc8b1*-rev: AGT​CAC​AGC​GAT​CAG​ATG​TGT, *18S-*for: CGG​CGA​CGA​CCC​ATT​CGA​AC, *18S*-rev: GAA​TCG​AAC​CCT​GAT​TCC​CCG​T.

### Western Blot

Cells were resuspended in RIPA lysis buffer with complete protease inhibitor cocktail (Thermo Scientific). 40 μg of total protein was fractionated by SDS-PAGE and transferred onto a nitrocellulose membrane. Membranes were incubated with antibodies against β-Actin (1:5,000, mouse, clone C4; SCBT), VDAC (1:1,000, mouse, N152B/23; BioLegend), GAPDH (1:200, mouse, 6C5; SCBT) and MCU (1:1,000, rabbit polyclonal; CST). For detection, peroxidase-coupled secondary anti-mouse or anti-rabbit antibodies (BioRad) and ECL Substrate (Thermo Scientific) were used.

### Metabolic Flux Analyses

Glycolytic proton efflux rate (PER) and oxygen consumption rate (OCR) were measured using a XFe96 extracellular flux analyzer (Seahorse Bioscience) as described before (Vaeth et al., 2017a; Kahlfuss et al., 2020).

### Statistical Analyses

All results are means with standard error of the means (SEM). The statistical significance of differences between experimental groups was determined by unpaired Student’s *t*-test. Differences were considered significant for p values < 0.05.

## Results

### Ablation of MCU Elevates Cytosolic Ca^2+^ Levels After Antigen Receptor Stimulation

**FIGURE 1:**
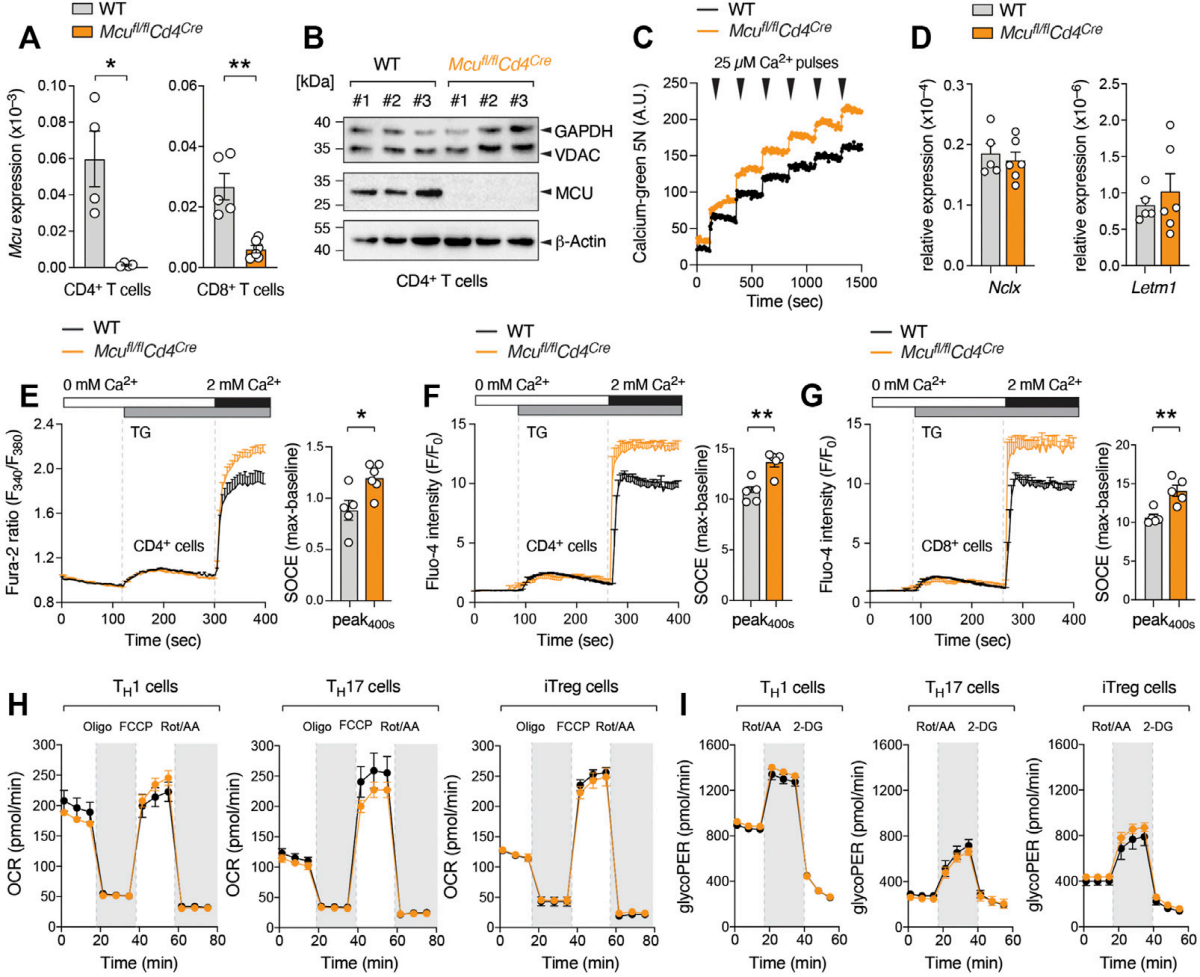
Deletion of MCU attenuates mitochondrial Ca^2+^ uptake but increases cytosolic Ca^2+^ levels after store depletion. **(A)** *Mcu* gene expression in CD4^+^ and CD8^+^ T cells of WT and MCU-deficient mice (*Mcu* ^fl/fl^ *Cd4* ^Cre^) analyzed by qRT-PCR; means ± SEM of 4-6 mice. **(B)** Immunoblot assay detecting MCU, VDAC and GAPDH protein expression in anti-CD3/CD28 stimulated CD4^+^ T cells, three independent WT and MCU-deficient mice. **(C)** Ca^2+^ retention capacity of digitonin-permeabilized WT and MCU-deficient T cells after repetitive addition of 25 µM extracellular Ca^2+^ measured by CalciumGreen-5N. **(D)** Analysis of *Slc8b1* (NCLX) and *Letm1* gene expression in activated WT and MCU-deficient CD8^+^ T cells by qRT-PCR; means ± SEM of 5-6 mice. **(E)** Measurement of Ca^2+^ store depletion and SOCE in CD4^+^ T cells isolated from WT and *Mcu* ^fl/fl^ *Cd4* ^Cre^ mice using a FlexStation3 plate reader. Cells were loaded with Fura-2 and stimulated with thapsigargin (TG) in Ca^2+^ free Ringer solution followed by re-addition of 2 mM Ca^2+^ **(left panel)**. The traces were baseline normalized and the quantification of maximal SOCE (peak-baseline) was calculated as F340/380 emission ratios; means ± SEM of 4-6 mice **(right panel)**. **(F, G)** *Ex vivo* analysis of store depletion and SOCE in CD4^+^ **(F)** and CD8^+^ **(G)** T cells of WT and *Mcu* ^fl/fl^ *Cd4* ^Cre^ mice loaded with Fluo-4 and analyzed by flow cytometry. Addition of TG and 2 mM extracellular Ca^2+^ as indicated. Baseline normalized (F/F_0_) means ± SEM of 4-5 mice. **(H, I)** Seahorse extracellular flux analyses to measure oxygen consumption rate (OCR) **(H)** and glycolytic proton efflux rate (glycoPER) **(I)** in WT and MCU-deficient Th1, Th17 and iTreg cells; means ± SEM of 3-5 mice.

To investigate the role of MCU (CCDC109A) and mitochondrial Ca^2+^ uptake in primary T cells, we generated mice with T cell-specific deletion of the *Mcu* gene by crossing *Mcu* ^fl/fl^ mice (Kwong et al., 2015) to *Cd4* ^Cre^ animals that express *Cre* recombinase under the control of the *Cd4* promoter. *Mcu* ^fl/fl^ *Cd4* ^Cre^ mice were indistinguishable from their littermates and showed a normal composition of thymic T cell populations (Supplementary Figure S1A). The frequencies and numbers of peripheral conventional and regulatory (Treg) T cells were also unaltered (Supplementary Figures S1B–D). Naïve and effector T cell composition was comparable between WT and *Mcu* ^fl/fl^ *Cd4* ^Cre^ mice and MCU-deficient mice showed no spontaneous immune dysregulation (Supplementary Figure S1E). We next confirmed that the expression of MCU was completely abolished in T cells at the mRNA (Figure 1A) and protein level (Figure 1B). We next measured mitochondrial Ca^2+^ uptake in digitonin-permeabilized WT and MCU-deficient T cells after extracellular Ca^2+^ addition (Figure 1C). Although we observed a reduced mitochondrial Ca^2+^ uptake in absence of MCU, primary mouse T cells showed only a moderate capacity to buffer extracellular Ca^2+^ elevations. Typically, the extracellular Ca^2+^ pulses are rapidly taken up by the mitochondria until mPTP opening becomes visible by a sudden elevation of free cytosolic Ca^2+^. Our best explanation why we did not measure significant mitochondrial Ca^2+^ uptake in these experiments is that primary T cells have fewer and relatively small mitochondria compared to other immune cells, such as macrophages, which makes it difficult to monitor their Ca^2+^ buffering capacity. In addition, other mitochondrial Ca^2+^ transporters, such as the Na^+^/Ca^2+^/Li^+^ exchanger NCLX and the H^+^/Ca^2+^ exchanger Letm1, may compensate for the loss of MCU. Although expression of NCLX and Letm1 was unchanged (Figure 1D), this data does not exclude adaptational changes in mitochondrial Ca^2+^ handling and other compensatory mechanisms, when MCU is genetically ablated.

Previous reports presented conflicting results regarding MCU’s effect on cytosolic Ca^2+^ elevations. In most studies, genetic or pharmacological inhibition of MCU attenuated ER store depletion and SOCE (Hoth et al., 1997; Gilabert et al., 2001; Samanta et al., 2014; Samanta et al., 2020), presumably due to a negative feedback regulation known as Ca^2+^-dependent inactivation (CDI) of IP_3_R and/or CRAC channels (Samanta et al., 2014; Deak et al., 2014; Tang et al., 2015). By contrast, other studies reported unchanged or elevated SOCE upon silencing of MCU (Paupe and Prudent, 2018; Wang P. et al., 2020; Seegren et al., 2020). In our experiments using primary MCU-deficient T cells, we observed a small but reproducible increase in cytosolic Ca^2+^ levels after ER store depletion with the sarco/endoplasmic reticulum Ca^2+^ ATPase (SERCA) inhibitor thapsigargin (TG) (Figures 1E–G). Although unexpected, our results are in line with recent data that also found that deletion of MCU by CRISPR/Cas9-mediated genome editing elevates SOCE and NFAT nuclear translocation in different lymphocytic cell lines and in primary T and B cells (Yoast et al., 2021).

### MCU is Dispensable for T Cell Proliferation, Differentiation and Effector Function *In Vitro*

**FIGURE 2:**
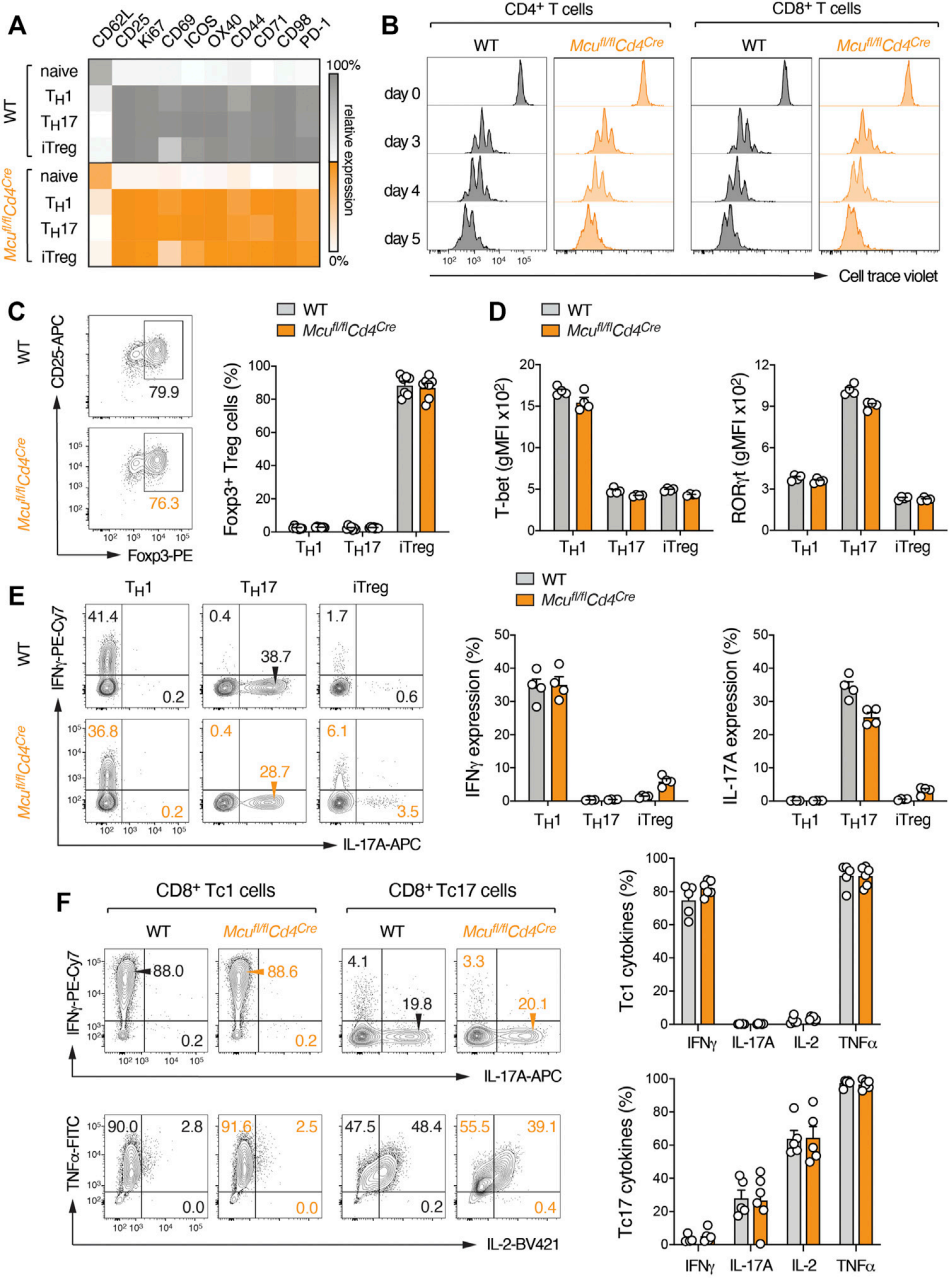
Ablation of MCU does not impair activation, differentiation and effector function of murine T cells *in vitro*. **(A)** Flow cytometric analysis of CD62L and activation marker (CD25, Ki-67, CD69, ICOS, OX-40, CD44, CD71, CD98 and PD-1) expression on WT and MCU-deficient T cells after anti-CD3/CD28 stimulation. Naive CD4^+^ T cells were cultured for 3 days under Th1, Th17 and iTreg culture conditions; heatmap showing row-normalized geometric MFIs as means of 3 individual mice. **(B)** Representative proliferation analysis of WT and MCU-deficient CD4^+^ and CD8^+^ T cells by CellTrace Violet dilution after anti-CD3/CD28 stimulation over the course of 5 days. **(C)** Analysis of Foxp3 expression in WT and MCU-deficient T cells cultured for 3 days under Th1, Th17 and iTreg**-**polarizing conditions; means ± SEM of 7 mice. **(D)** Flow cytometric analyses of T-bet and RORγt expression in WT and MCU-deficient T cells cultured under Th1, Th17 and iTreg-polarizing conditions; means ± SEM of 4 mice. **(E)** Quantification of IFNγ and IL-17A protein expression in WT and MCU-deficient CD4^+^ T cells differentiated into Th1, Th17 and iTreg cells for 3 days and re-stimulated with PMA/ionomycin for 5 h; means ± SEM of 4 mice. **(F)** Quantification of IFNγ, IL-17A, IL-2 and TNFα production by WT and MCU-deficient CD8^+^ T cells differentiated into Tc1 and Tc17 cells for 6 days and re-stimulated with PMA/ionomycin for 5 h; means ± SEM of 5-6 mice.

To determine how impaired mitochondrial Ca^2+^ buffering and elevated SOCE affects the activation and effector function of T cells, we isolated naïve CD4^+^ T cells from WT and *Mcu* ^fl/fl^ *Cd4* ^Cre^ mice and differentiated these cells into different T helper (Th) subsets. The expression of MCU was similar in all Th cells tested (Supplementary Figure S2A). We focused on Th1, Th17 and iTreg cells for further analyses as these subsets are not only well-defined by their (inflammatory) cytokine profiles but also differ in their dependency on mitochondrial metabolism. However, we did not detect differences in oxygen consumption rate (OCR) or extracellular acidification (glycoPER) as measures of mitochondrial and glycolytic activity, respectively, when we compared MCU-deficient T cells to WT controls (Figures 1H,I). Stimulation of WT and MCU-deficient T cells under Th1, Th17 and iTreg-polarizing conditions revealed also no difference in the expression of activation markers (Figure 2A). Cell cycle entry (Supplementary Figure S2B) and proliferation of MCU-deficient CD4^+^ and CD8^+^ T cells was also comparable to WT controls (Figure 2B), indicating that MCU is dispensable for T cell metabolism and activation. To test whether MCU affects T cell differentiation, we analyzed the expression of T-bet, RORγt and Foxp3 as the “signature” transcription factors of Th1, Th17 and iTreg cells, respectively. We observed, however, no differences in the generation of iTreg (Figure 2C) or inflammatory Th1 and Th17 cells *in vitro* (Figure 2D, Supplementary Figure S2C), suggesting that mitochondrial Ca^2+^ is not required for T cell differentiation. Finally, we tested whether MCU plays a role in the effector function of T cells by re-stimulating Th1, Th17 and iTreg cells with PMA and ionomycin and analyzed the cytokine expression of WT and MCU-deficient cells by flow cytometry. However, we did not observe significant differences in IFNγ and IL-17A expression by Th1 and Th17 cells, respectively (Figure 2E). Likewise, GM-CSF and IL-2 expression was unaltered in MCU-deficient T cells (Supplementary Figure S2D). Similar as “helper” CD4^+^ T cells, “cytotoxic” CD8^+^ T cells can be also polarized into Tc1 and Tc17 cells that are not only characterized by an inflammatory (Tc1) and memory (Tc17) phenotype but also differ greatly by their mitochondrial activity (Flores-Santibáñez et al., 2018). As in CD4^+^ T cells, ablation of MCU did not affect differentiation and cytokine expression of Tc1 and Tc17 cells (Figure 2F). Collectively, these data suggest that genetic deletion of MCU does not alter T cell metabolism, differentiation and effector function *in vitro*. By contrast, the widely used, but unspecific, MCU inhibitor Ruthenium Red attenuated T cell metabolism, proliferation and survival (Supplementary Figures S3A–F). Because pharmacological blockade of MCU does not recapitulate the findings of MCU-deficient T cells, this data hints at compensatory mechanisms in MCU knockout mice and/or off-target effects of pharmacological inhibitors.

### Loss of MCU Does not Affect Adaptive Immune Responses in Autoimmunity and Infection

**FIGURE 3:**
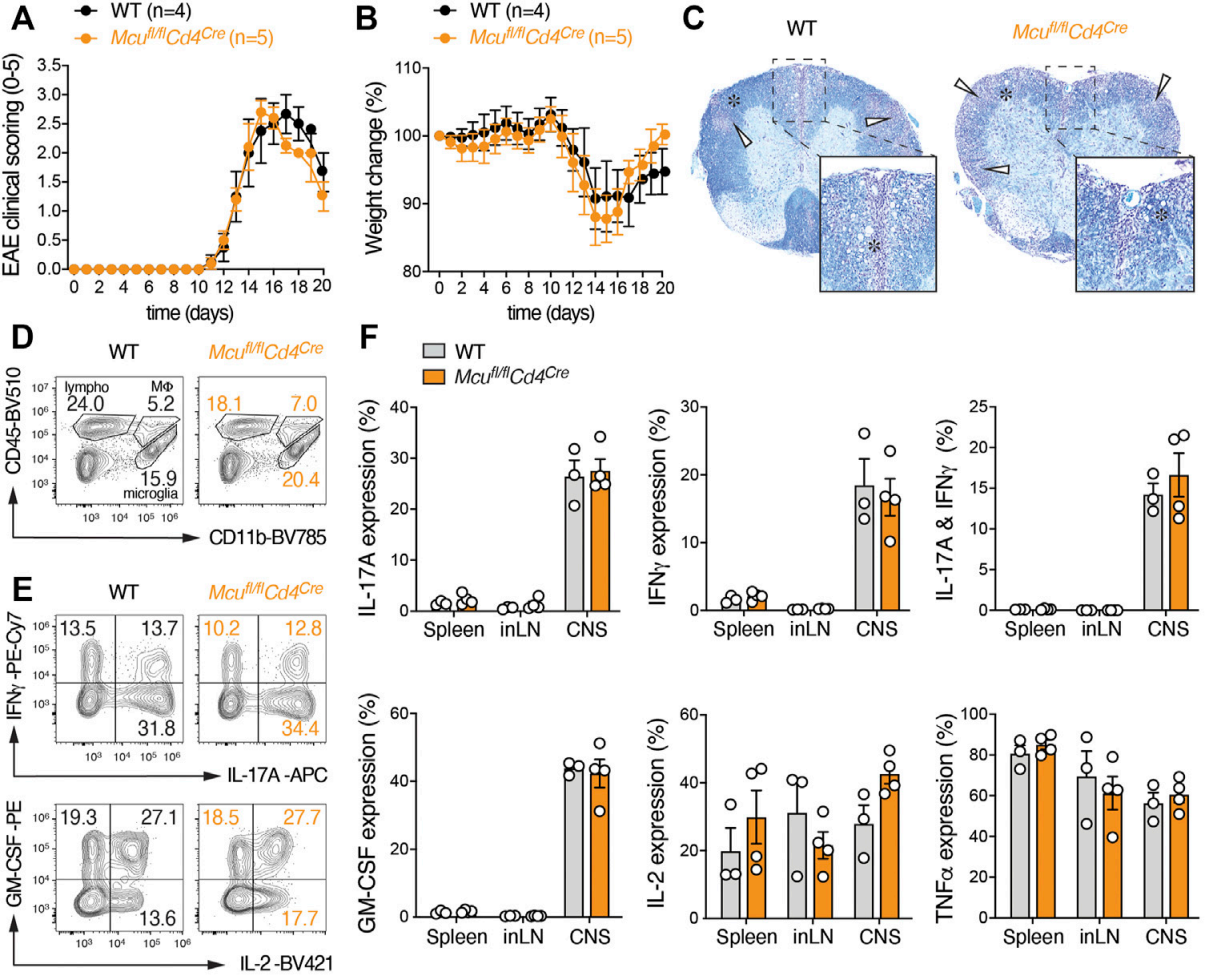
Mice with T cell-specific deletion of MCU are susceptible to experimental autoimmune encephalomyelitis (EAE). **(A)** Clinical EAE scores of WT and MCU-deficient (*Mcu* ^fl/fl^ *Cd4* ^Cre^) mice after immunization with MOG_35-55_ peptide; means ± SEM of 4-5 mice per cohort. **(B)** Relative weight change of WT and MCU-deficient mice after immunization with MOG_35-55_ peptide emulsified in CFA; means ± SEM of 4-5 mice per cohort. **(C)** Histopathological examination of spinal cord sections of WT and MCU-deficient mice 20 days after MOG_35-55_ immunization stained with Luxol fast blue (myelin) and Cresyl violet (nuclei). White arrows indicate leukocytic infiltrates and areas of demyelination (denoted with an asterisk). **(D)** Analyses of lymphocytic infiltrations in the CNS of WT and MCU-deficient mice 20 days after MOG_35-55_ immunization. **(E)** Flow cytometric analyses of IFNγ, IL-17A, IL-2 and GM-CSF production of CD4^+^ T cells isolated from the CNS of WT and *Mcu* ^fl/fl^ *Cd4* ^Cre^ mice 20 days after MOG_35-55_ peptide immunization and stimulation with PMA/Iono for 5 h. **(F)** Frequencies of IL-17A, IFNγ, GM-CSF, IL-2 and TNFα-producing CD4^+^ T cells in the spleen, inLN and CNS of MOG_35-55_-imunized WT and *Mcu* ^fl/fl^ *Cd4* ^Cre^ mice after stimulation with PMA/ionomycin for 5 h; means ± SEM of 3-4 mice.

**FIGURE 4:**
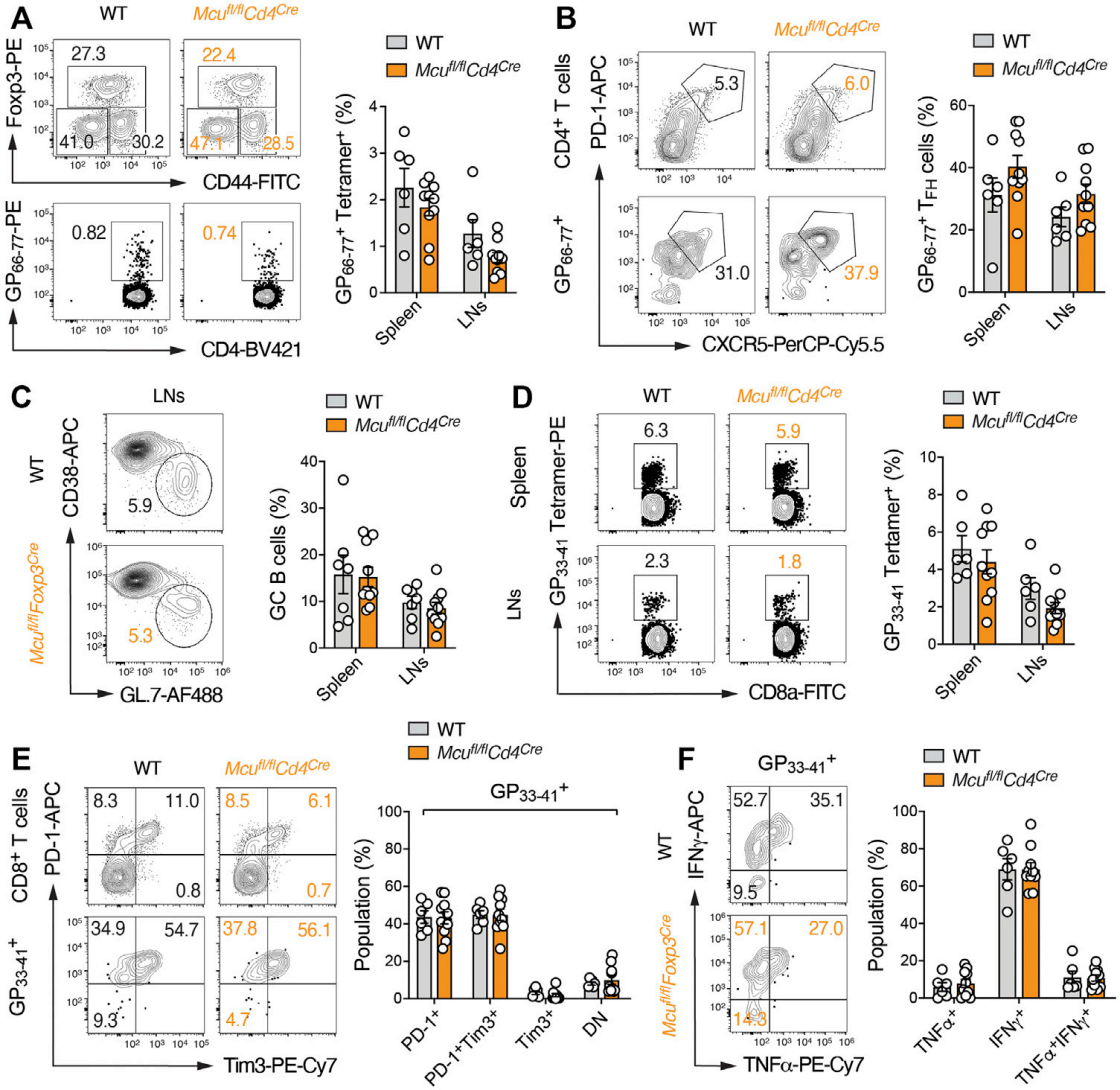
T cell-specific ablation of MCU does not affect adaptive immune responses to persistent LCMV infection. **(A**–**C)** CD4^+^ T cell immune responses in chronically infected WT and MCU-deficient (*Mcu* ^fl/fl^ *Cd4* ^Cre^) mice. **(A)** Analysis of Foxp3^+^ Treg and LCMV-specific (GP_66-77_ tetramer^+^) effector CD4^+^ T cell populations in WT and MCU-deficient mice 23 days after LCMV infection; means ± SEM of 6–10 mice. **(B)** Flow cytometric analysis of T follicular helper (T_FH_) cells in total CD4^+^ and LCMV-specific (GP_66-77_ tetramer^+^) T cells 23 days after LCMV infection; means ± SEM of 6–10 mice. **(C)** Quantification of germinal center (GC) B cells in spleen and LNs of WT and *Mcu* ^fl/fl^ *Cd4* ^Cre^ mice; means ± SEM of 6–10 mice. **(D**–**F)** CD8^+^ T cell immune responses in chronically infected WT and MCU-deficient mice. **(D)** Flow cytometric quantification of LCMV-specific (GP_33-41_ tetramer^+^) effector CD8^+^ T cell populations in the spleens and LNs of WT and MCU-deficient mice 23 days after infection; means ± SEM of 6-9 mice. **(E)** Analysis of PD-1 and Tim3 expression on total CD8^+^ and LCMV-specific (GP_66-77_ tetramer^+^) T cells 23 days after LCMV infection; means ± SEM of 6–10 mice. **(F)** Frequencies of IFNγ and TNFα-producing LCMV-specific (GP_33-41_ tetramer^+^) T cells in WT and *Mcu* ^fl/fl^ *Cd4* ^Cre^ mice 10 d post infection and re-stimulation with PMA/ionomycin for 5 h; means ± SEM of 6-9 mice.

Although *in vitro* assays are useful to evaluate basic functions of T cells, the complexity of T cell interaction with lymphoid and non-lymphoid cell types and the varying metabolic conditions in different target tissues cannot be fully emulated *in vitro*. We therefore employed two complementary animal models of T cell-mediated inflammation; experimental autoimmune encephalomyelitis (EAE) (Figure 3), which resembles aspects of human multiple sclerosis, and persistent viral infection with the lymphocytic choriomeningitis virus (LCMV) strain clone 13 (Figure 4). We first induced EAE in *Mcu* ^fl/fl^ *Cd4* ^Cre^ and littermate control mice by immunization with MOG_35-55_ peptide emulsified in CFA and monitored the disease progression over the course of 20 days. T cell-specific deletion of MCU did not alter EAE immunopathology, including the paralysis of their extremities (Figure 3A), inflammation-induced weight loss (Figure 3B) and the demyelination of the spinal cord (Figure 3C). The infiltration of lymphocytes into the CNS was also similar in *Mcu* ^fl/fl^ *Cd4* ^Cre^ mice compared to control animals (Figure 3D). In line with our *in vitro* data, the production of IFNγ, IL-17A, GM-CSF, IL-2 and TNFα by encephalitogenic Th1 and Th17 cells following re-stimulation with PMA and ionomycin was not impaired in absence of MCU (Figures 3E,F). Because mitochondrial Ca^2+^ uptake is impaired in all T cells of *Mcu* ^fl/fl^ *Cd4* ^Cre^ mice, defects in both inflammatory and regulatory T cells could potentially mask subset-specific functions of MCU. To explore a cell-intrinsic role of MCU in Treg cells, we generated *Mcu* ^fl/fl^ *Foxp3* ^Cre^ mice that lack MCU expression specifically in Foxp3^+^ Treg cells. As observed in mice with ablation of MCU in all T cells, *Mcu* ^fl/fl^ *Foxp3* ^Cre^ mice were indistinguishable from their littermates and showed normal frequencies of thymocytes and peripheral T cells (Supplementary Figure S4A–D). Importantly, *Mcu* ^fl/fl^ *Foxp3* ^Cre^ mice showed no signs of an overt immune activation (Supplementary Figure S4E) and the numbers of Treg cells in peripheral lymphoid organs were unaltered. The differentiation of thymus-derived Treg cells into CD44^+^CD62L^–^ effector Treg cells was also not perturbed in absence of MCU (Supplementary Figures S4F,G), suggesting that MCU is not required for Treg development and their suppressive function *in vivo*. In addition to autoimmunity and T cell responses to self-antigens, we tested the anti-viral activity of CD4^+^ and CD8^+^ T cells in WT and *Mcu* ^fl/fl^ *Cd4* ^Cre^ mice after infection with the LCMV strain clone 13 (Figure 4). LCMV infection promotes the differentiation of CD4^+^ T cells into T follicular helper (Tfh) cells to support affinity maturation of germinal center (GC) B cells and anti-viral humoral immunity. We did not detect differences in the activation of CD4^+^ T cells and Foxp3^+^ Treg cells in *Mcu* ^fl/fl^ *Cd4* ^Cre^ mice compared to WT controls (Figure 4A). The clonal expansion of LCMV-specific CD4^+^ T cells, which were monitored with tetramers loaded with the LCMV-derived GP_66-77_ peptide, was also unaltered (Figure 4A). Furthermore, Tfh cell differentiation (Figure 4B) and GC B cell responses (Figure 4C) were intact in *Mcu* ^fl/fl^ *Cd4* ^Cre^ mice, demonstrating that MCU is dispensable for ‘helper’ T cell responses during viral infection. Although LCMV induces a strong CD8^+^ T cell-mediated immune response, persistent antigenic stimulation during chronic LCMV infection also promotes T cell ‘exhaustion’ that is characterized by an upregulation of inhibitory receptors (such as PD-1 and Tim3), loss of effector function (e.g., cytokine expression) and apoptosis of CD8^+^ T cells (McLane et al., 2019). Using GP_33-41_ tetramers to identify LCMV-specific CD8^+^ T cells, we did not observe differences in the generation and/or expansion of cytotoxic immune responses in *Mcu* ^fl/fl^ *Cd4* ^Cre^ mice compared to control animals (Figure 4D). T cell exhaustion (Figure 4E) and cytokine expression (Figure 4F) of MCU-deficient T cells were also comparable to those in WT mice, suggesting that MCU is also expendable for ‘cytotoxic’ anti-viral immune responses.

Collectively, these data demonstrate that, despite attenuating mitochondrial Ca^2+^ uptake and elevating cytosolic Ca^2+^ levels after antigen receptor stimulation, MCU is largely dispensable for T cell-mediated immune responses *in vitro* and in models of autoimmunity and persistent viral infection.

## Discussion

Mitochondrial Ca^2+^ uptake is crucial for many cellular functions, including oxidative metabolism, ROS production, signal transduction and the regulation of cell death. In stressed or chronically activated cells, MCU triggers mitochondrial Ca^2+^ overload that causes the opening of the mPTP and the release of pro-apoptotic factors. Thus, tight control of mitochondrial Ca^2+^ uptake safeguards cellular survival and alterations in mitochondrial Ca^2+^ handling have been linked to a variety of diseases. Not surprisingly, MCU gained attention as a target for therapeutic interventions in different pathologies, including cancer, cardiovascular, and inflammatory disorders.

Ca^2+^ signals play fundamental roles in the immune system. They not only control the differentiation and effector function of lymphocytes at the transcriptional level but also promote their metabolic re-programming (Vaeth et al., 2020). We have recently shown that SOCE regulates multiple metabolic pathways in T cells, including glycolysis, mitochondrial metabolism and OXPHOS (Vaeth et al., 2017a; Wang Y. et al., 2020; Kahlfuss et al., 2020). SOCE-mediated NFAT signaling induces the expression of glucose transporters and glycolytic enzymes, resulting in an impaired glucose uptake and metabolism when extracellular Ca^2+^ influx or NFAT is inhibited (Vaeth et al., 2017a; Vaeth et al., 2020). The regulation of mitochondrial metabolism by SOCE is, however, less well understood (Wang Y. et al., 2020). Ca^2+^ influx through SOCE triggers MCU opening and the uptake of cytosolic Ca^2+^ into the mitochondrial matrix could directly stimulate OXPHOS and mitochondrial ATP synthesis. On the other hand, many SOCE-dependent mitochondrial proteins are encoded in the nucleus, arguing that cytosolic Ca^2+^ could also control mitochondrial metabolism independently of MCU.

We here show, contrary to our expectation, that ablation of MCU has no obvious effects on T cell metabolism, differentiation and effector function *in vitro* and in animal models of autoimmunity and viral infection. Although our results were unexpected, they are in line with the unaltered or mild phenotypes of mice with global or conditional deletion of MCU in various non-immune tissues (Wang P. et al., 2020). Of note, all published MCU-deficient mouse strains did not reveal an energy crisis, suggesting MCU-mediated Ca^2+^ uptake is largely dispensable for mitochondrial metabolism under basal conditions. Only after acute stimulation and/or stress of tissues with a high mitochondrial workload, such as skeletal or cardiac muscle, genetic inhibition of mitochondrial Ca^2+^ uptake revealed moderate effects, albeit not consistently observed in all MCU-deficient strains (Wang P. et al., 2020). The findings presented in this study and previous reports using MCU-deficient mice are in stark contrast to the expected importance of mitochondrial Ca^2+^ uptake that was predicted from *in vitro* experiments. This discrepancy may be explained by the presence of additional, so far undefined, mitochondrial Ca^2+^ handling molecules or other (ionic) adaptions that compensate for the lack of MCU *in vivo* (Wang P. et al., 2020).

The consequences of mitochondrial Ca^2+^ uptake on cytosolic Ca^2+^ levels remain complex (Paupe and Prudent, 2018; Wang P. et al., 2020; Yoast et al., 2021). In most previous reports, MCU blockade by pharmacological inhibition or RNAi attenuated ER store depletion and SOCE (Hoth et al., 1997; Gilabert et al., 2001; Deak et al., 2014; Samanta et al., 2014; Tang et al., 2015; Samanta et al., 2020). Other studies found that MCU-deficient lymphocytes and macrophages showed enhanced store depletion and/or SOCE (Seegren et al., 2020; Yoast et al., 2021). Impaired cytosolic Ca^2+^ influx in absence of MCU was explained by an accelerated CDI of IP_3_R and/or CRAC channels that causes their premature closing when incoming Ca^2+^ cannot be taken up by adjacent mitochondria (Quintana et al., 2007; Lioudyno et al., 2008; Quintana et al., 2011; Deak et al., 2014; Samanta et al., 2014; Tang et al., 2015). In this study, we found that genetic ablation of MCU did not impair store depletion and SOCE but, instead, caused a slightly enhanced extracellular Ca^2+^ influx in primary T cells. These observations are in line with recent reports of macrophages and lymphocytes from MCU-deficient mice that showed a similar increase of cytosolic Ca^2+^ (Seegren et al., 2020; Yoast et al., 2021). Furthermore, complete deletion of mitochondria in activated T cells enhanced SOCE (Lisci et al., 2021), indicating that mitochondria *per se* and, thus, mitochondrial Ca^2+^ buffering are not essential for SOCE in primary T cells. Our seemingly contradictory findings compared to other reports may have a simple explanation: defective Ca^2+^ uptake from the cytosol into the mitochondrial matrix in MCU-deficient lymphocytes causes an accumulation of cytoplasmic Ca^2+^, resulting in a net increase of SOCE. Enhanced SOCE in MCU-deficient T cells could have been expected to amplify and augment T cell effector function and metabolism, which was, however, not the case. These observations are reminiscent of our previous findings using ORAI2-deficient mice that show a similar elevation of SOCE without increasing the effector functions of T cells in animal models of infection and autoimmunity (Vaeth et al., 2017b).

A possible explanation for our finding that MCU is largely dispensable for murine T cell function is that adaptational changes in the mitochondria may compensate for the “chronic” loss of MCU in *Mcu* ^fl/fl^ *Cd4* ^Cre^ mice. It was shown before that deletion of MCU alone is not sufficient to completely abolish mitochondrial Ca^2+^ uptake in neurons (Hamilton et al., 2018). Furthermore, mitochondria store large amounts of Ca^2+^ as precipitates and acidification of the mitochondrial matrix during respiration dissolves these crystals and releases intra-mitochondrially free Ca^2+^ ions in an MCU-independent fashion (Hernansanz-Agustín et al., 2020). Although we did not find significant effects of MCU on T cell proliferation, differentiation and effector function, our results do not exclude a potential role of MCU in the adaptive immune system. It is noteworthy that we could not test all scenarios in which T cells play an important role and that MCU’s importance could be different in human T cells. Our findings also have important technical implications as previous studies relied frequently on pharmacological MCU inhibitors but the genetic deletion of MCU does not recapitulate these findings. However, Ruthenium-based inhibitors are taken up inefficiently by intact cells, have a poor selectivity for MCU and affect, in addition, other Ca^2+^ channels, such as ryanodine receptors, TRP channels and SERCA pumps (Finkel et al., 2015; Woods and Wilson, 2020). Thus, future studies using genetic approaches are warranted to better define MCU’s role in murine and human T cells and other (non-)immune cell types.

## Data Availability

The raw data supporting the conclusions of this article will be made available by the authors, without undue reservation.
