# Supplementary material for: Genetic Ablation of the Mitochondrial Calcium Uniporter (MCU) Does not Impair T Cell-Mediated Immunity In Vivo
Source: Front Pharmacol. 2021 Dec 20;12:734078. doi: 10.3389/fphar.2021.734078 (PMC8721163; doi:10.3389/fphar.2021.734078)
Supplement: Supplementary file 1 [file DataSheet1.PDF]

Supplementary Material

Genetic ablation of the mitochondrial calcium uniporter (MCU) does not impair T cell-mediated immunity *in vivo*

Hao Wu[1,4], Benjamin Brand[1,4], Miriam Eckstein[1], Sophia Hochrein[1], Magdalena Shumanska[2], Jan Dudek[3], Alexander Nickel[3], Christoph Maack[3], Ivan Bogeski[2], Martin Vaeth[1*]

[1]Institute for Systems Immunology, Julius-Maximilians University of Würzburg, Würzburg, Germany

[2]Molecular Physiology, Institute of Cardiovascular Physiology, University Medical Center, Georg-August-University, Göttingen, Germany

[3]Comprehensive Heart Failure Center, University Hospital, Julius-Maximilians University of Würzburg, Würzburg, Germany

[4]These authors have contributed equally to this work

*** Correspondence:**
Martin Vaeth, PhD
Martin.vaeth1@uni-wuerzburg.de

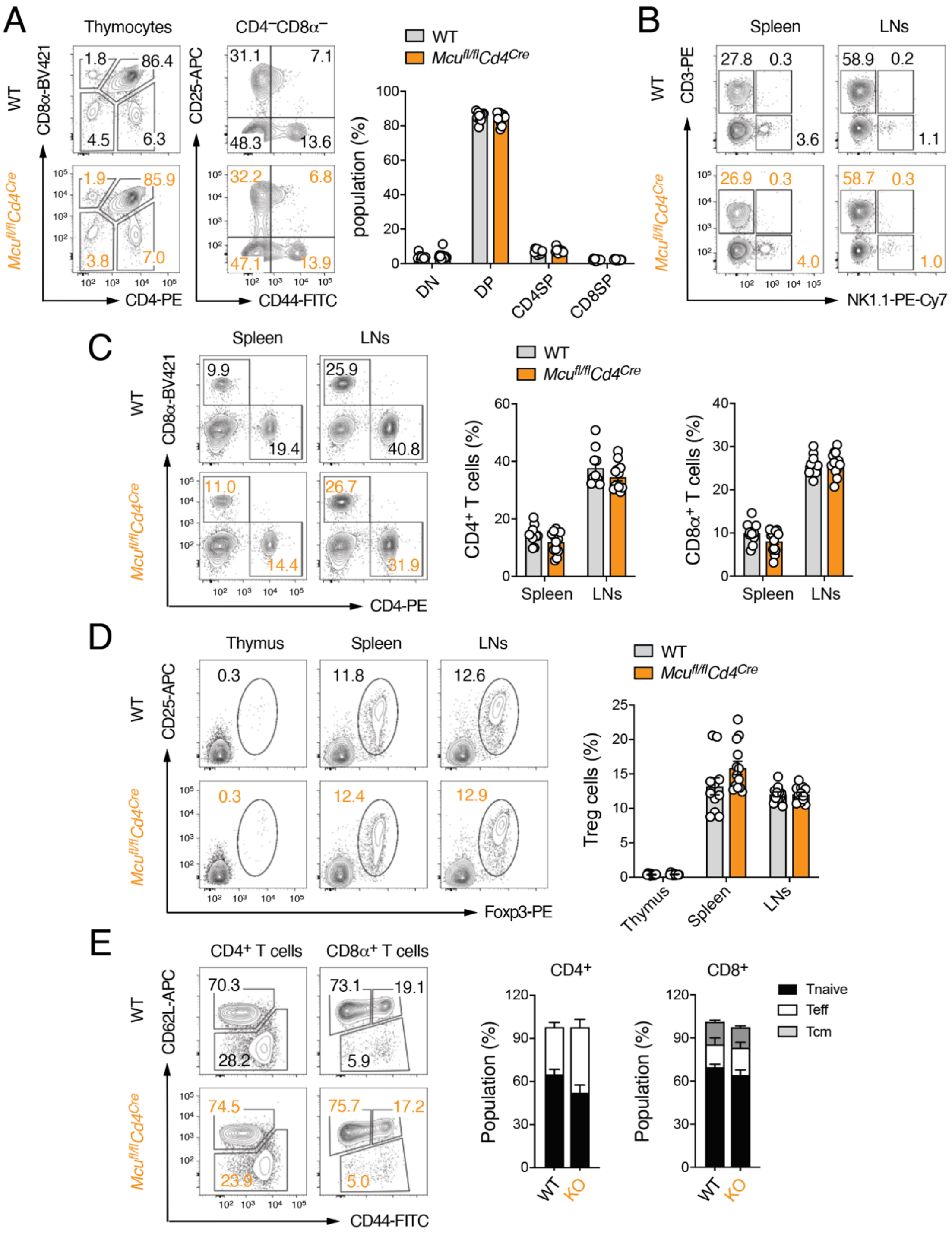

Supplementary Figure 1. Phenotype of mice with T cell-specific ablation of MCU. (A) Development of T cells in WT and *Mcu*$^{fl/fl}$*Cd4*^{Cre} mice. Flow cytometric analyses of CD4^{-}CD8^{-} (DN), CD4^{+}CD8^{+} (DP) and single positive (SP) thymocytes of WT and *Mcu*$^{fl/fl}$*Cd4*^{Cre} mice; means ± SEM of 11-13 mice. **(B-E)** Analysis of peripheral T cell subsets in WT and *Mcu*$^{fl/fl}$*Cd4*^{Cre} mice. Flow cytometric analyses of CD3^{+} and NKT **(B),** CD4^{+} and CD8^{+} **(C)**, and Foxp3^{+} Treg cells **(D)** in the spleens and LNs of WT and *Mcu*$^{fl/fl}$*Cd4*^{Cre} mice; means ± SEM of 11-13 mice. **(E)** Quantification of CD44^{-}CD62L^{+} (naïve), CD44^{+}CD62L^{+} (central memory) and CD44^{+}CD62L^{-} (effector) CD4^{+} and CD8^{+} T cells in the LNs of WT and MCU-deficient mice by flow cytometry; means ± SEM of 11-13 mice.

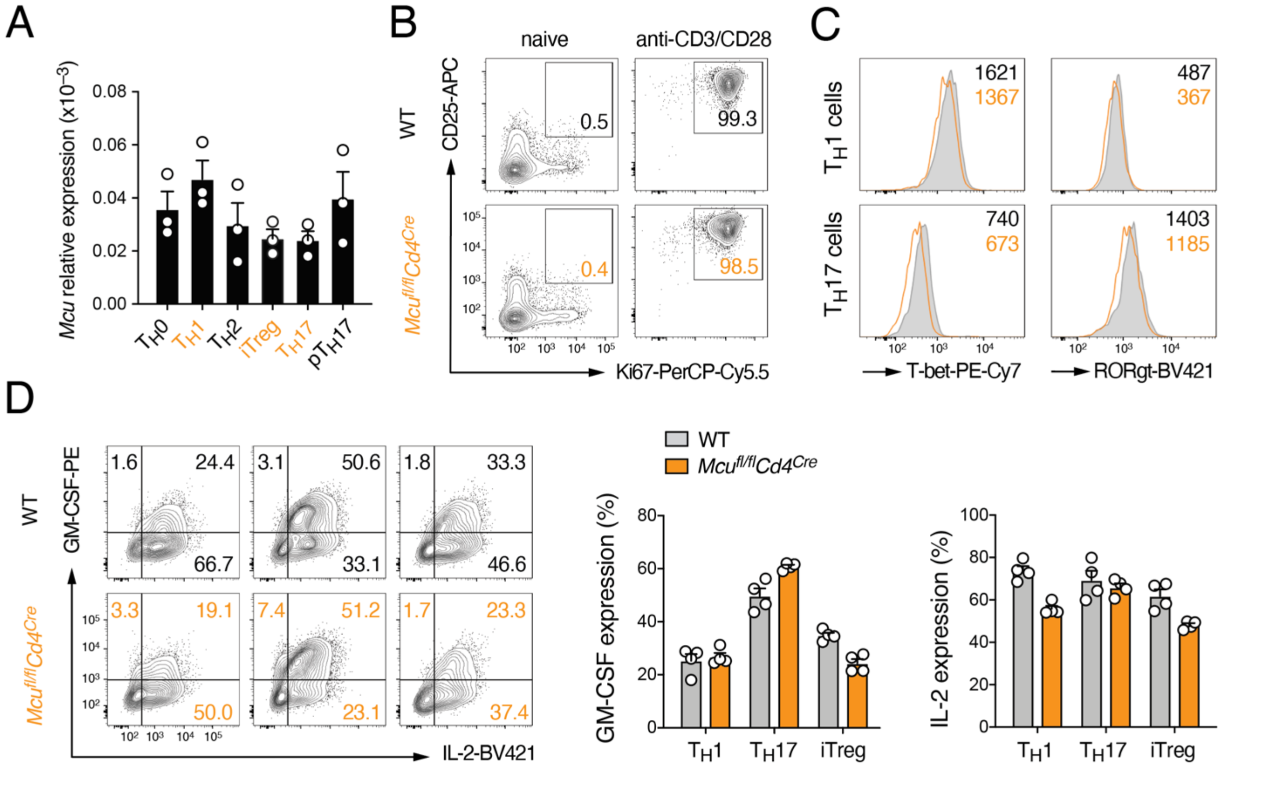

Supplementary Figure 2. Genetic ablation of MCU does not affect T cell differentiation *in vitro*. (A) Analysis of *Mcu* gene expression in WT CD4+ T cells after differentiation into Th0, Th1, Th2, Th17 and iTreg cells for 3 days by qRT-PCR; means ± SEM of 3 mice. **(B)** Analysis of CD25 and Ki-67 expression of naïve and anti-CD3/CD28 activated WT and MCU-deficient (*Mcu*$^{fl/fl}$*Cd4*^{Cre}) T cells. **(C)** Representative flow cytometric analyses of T-bet and RORγt expression in WT and MCU-deficient CD4+ T cells cultured for 3 days under Th1 and Th17 cell-polarizing conditions; numbers within the histograms represent geometric mean of fluorescence intensities (gMFI). **(D)** Quantification of GM-CSF and IL-2 production of WT and MCU-deficient CD4+ T cells differentiated into Th1, Th17 and iTreg cells for 3 days and re-stimulation with PMA/ionomycin for 5 h; means ± SEM of 4 mice.

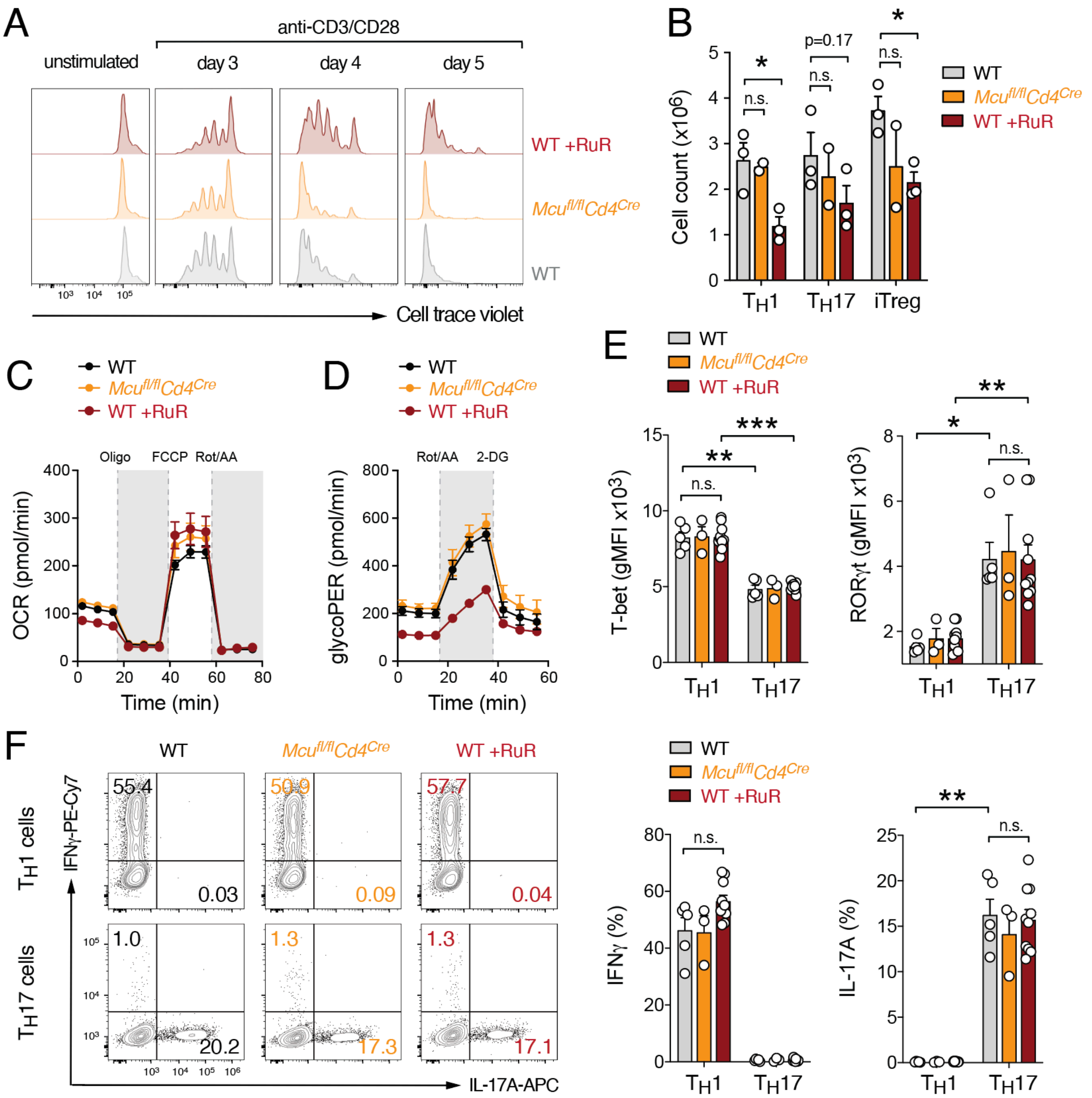

Supplementary Figure 3. Direct comparison of genetic and pharmacological MCU suppression in primary T cells. (A) Representative proliferation analysis of WT, MCU-deficient and Ruthenium Red-treated (RuR) $CD4^+$ T cells by CellTrace Violet dilution. **(B)** Cell counts of WT, MCU-deficient and RuR-treated Th1, Th17 and iTreg cells after 4 days of culture; means ± SEM of 2-3 mice. **(C, D)** Seahorse extracellular flux analyses measuring oxygen consumption rate (OCR) **(C)** and glycolytic proton efflux rate (glycoPER) **(D)** in WT, MCU-deficient and RuR-treated Th17 cells; means ± SEM of 3-5 mice. **(E)** Flow cytometric analyses of T-bet and RORγt expression in WT, MCU-deficient and RuR-treated $CD4^+$ T cells cultured under Th1 and Th17-polarizing conditions; means ± SEM of 3-5 mice. **(F)** Quantification of IFNγ and IL-17A expression in WT, MCU-deficient and RuR-treated Th1 and Th17 cells after re-stimulation with PMA/ionomycin for 5 h; means ± SEM of 3-5 mice.

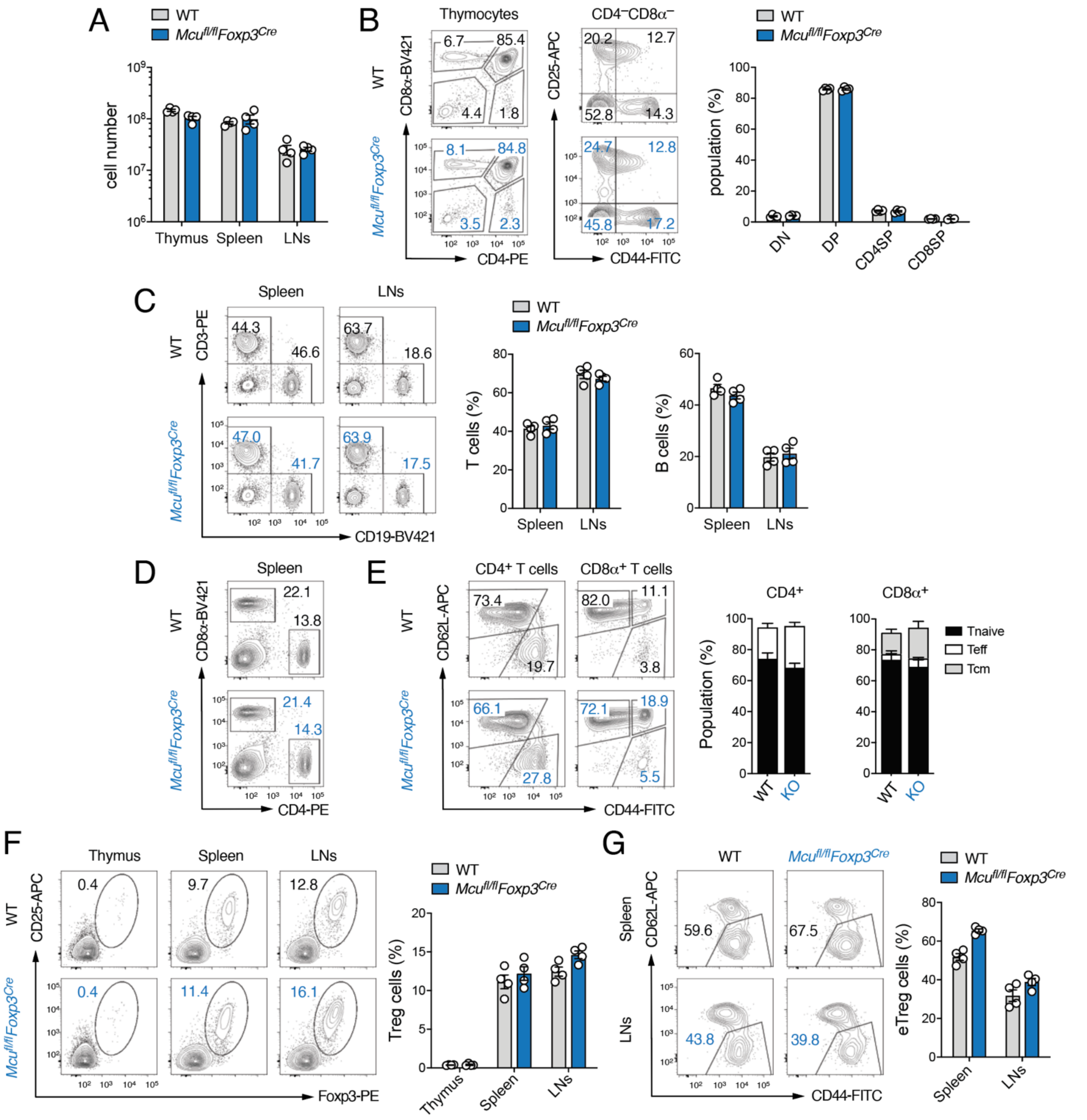

Supplementary Figure 4. Treg-specific deletion of MCU in *Mcu*$^{fl/fl}$*Foxp3*^{Cre} mice does not cause spontaneous immune activation. (A) Total cell numbers of thymus, spleen and LNs of WT and *Mcu*$^{fl/fl}$*Foxp3*^{Cre} mice; means ± SEM of 4 mice. **(B)** Development of T cells in WT and *Mcu*$^{fl/fl}$*Foxp3*^{Cre} mice. Representative flow cytometric analyses of $CD4^{-}CD8^{-}$ (DN), $CD4^{+}CD8^{+}$ (DP) and single positive (SP) thymocytes of WT and *Mcu*$^{fl/fl}$*Foxp3*^{Cre} mice; means ± SEM of 4 mice. **(C)** Analysis of peripheral lymphocyte subsets in WT and MCU-deficient mice. **(D)** Flow cytometric quantification of $CD4^{+}$ and $CD8^{+}$ T cells in the spleens and LNs of WT and *Mcu*$^{fl/fl}$*Foxp3*^{Cre} mice. **(E)** Analysis of $CD44^{-}$ $CD62L^{+}$ (naïve), $CD44^{+}CD62L^{+}$ (central memory) and $CD44^{+}CD62L^{-}$ (effector) T cells by flow cytometry; means ± SEM of 6-8 mice. **(F)** Frequencies of $Foxp3^{+}$ Treg cells in thymus, spleen and LNs of WT and *Mcu*$^{fl/fl}$*Foxp3*^{Cre} mice; means ± SEM of 4 mice. **(G)** Analysis of $CD44^{+}CD62L^{-}$ effector Treg (eTreg) cells in the spleens and LNs of WT and *Mcu*$^{fl/fl}$*Foxp3*^{Cre} mice; means ± SEM of 4 mice.
